# Supplementary material for: LIPL-1 and LIPL-2 are TCER-1-regulated Lysosomal Lipases with Distinct Roles in Immunity and Fertility
Source: bioRxiv. 2025 Jul 18:2025.07.14.664648. Preprint. [Version 1] doi: 10.1101/2025.07.14.664648 (PMC12338661; doi:10.1101/2025.07.14.664648)
Supplement: Supplement 1 [file NIHPP2025.07.14.664648v1-supplement-1.pdf]

FIGURE S1

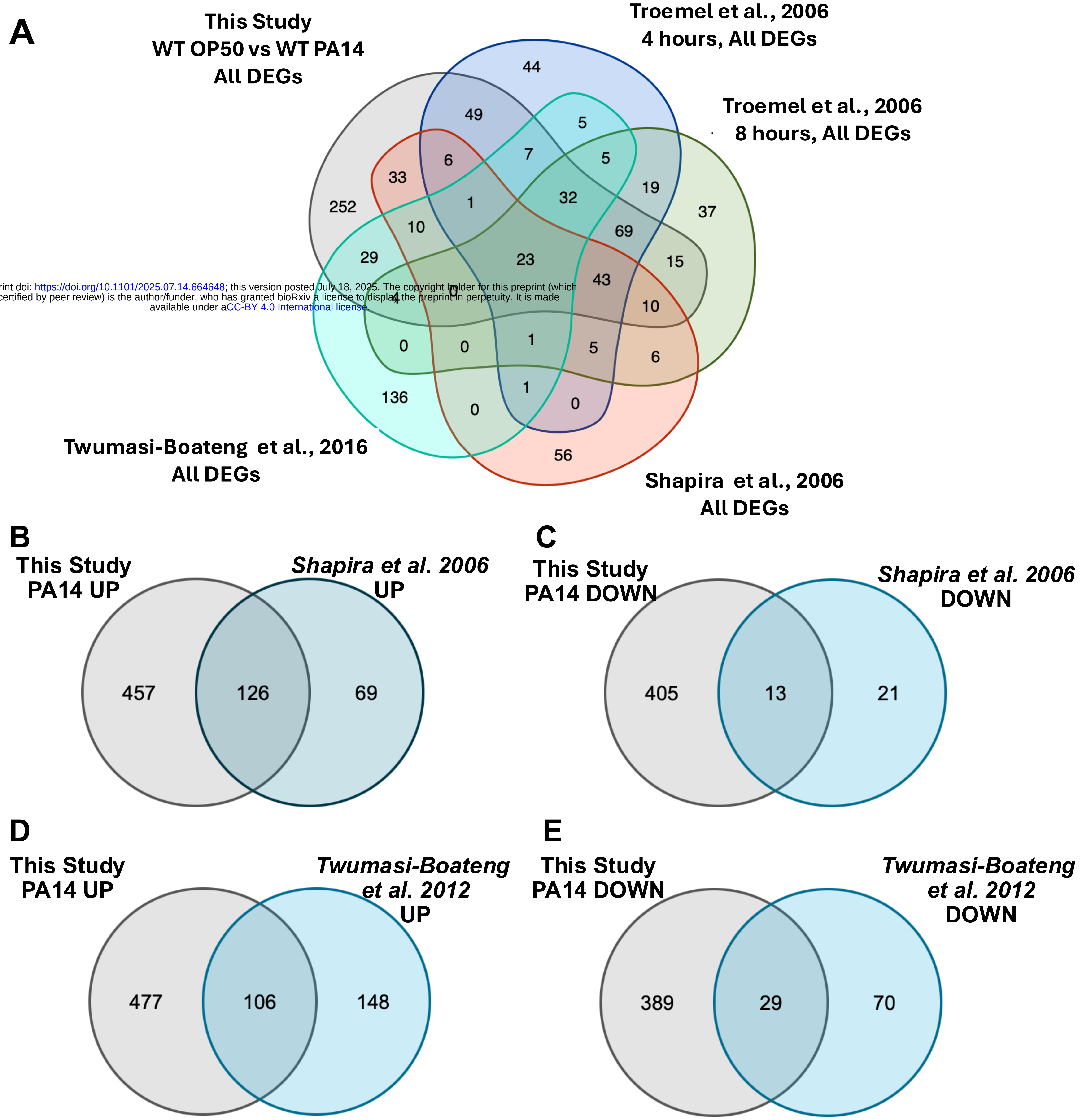

bioRxiv preprint doi: https://doi.org/10.1101/2025.07.14.664648; this version posted July 18, 2025. The copyright holder for this preprint (which was not certified by peer review) is the author/funder, who has granted bioRxiv a license to display the preprint in perpetuity. It is made available under aCC-BY 4.0 International license.

bioRxiv preprint doi: https://doi.org/10.1101/2025.07.14.664648; this version posted July 18, 2025. The copyright holder for this preprint (which was not certified by peer review) is the author/funder, who has granted bioRxiv a license to display the preprint in perpetuity. It is made available under aCC-BY 4.0 International license.

Supplementary Fig. S1: Overlap between PA14-induced differentially-expressed genes (DEGs) identified in this study and previously reported gene lists.

A: All genes up- and down-regulated upon normal animals' exposure to PA14 for 8 hours in this study compared with genes identified by Troemel et al. after 4 hours and 8 hours of exposure. **B- E:** Comparisons of DEGs upregulated **(B, D)** or downregulated **(C, E)** on PA14 with genes identified by Shapria et al., 2006 **(B, C)** and Twumasi-Boateng et al. 2012 **(D, E).** RF: Representation Factor. Statistical significance of overlap between gene sets calculated using hypergeometric probability formula with normal approximation (see Methods).

bioRxiv preprint doi: https://doi.org/10.1101/2025.07.14.664648; this version posted July 18, 2025. The copyright holder for this preprint (which was not certified by peer review) is the author/funder, who has granted bioRxiv a license to display the preprint in perpetuity. It is made available under aCC-BY 4.0 International license.

FIGURE S2

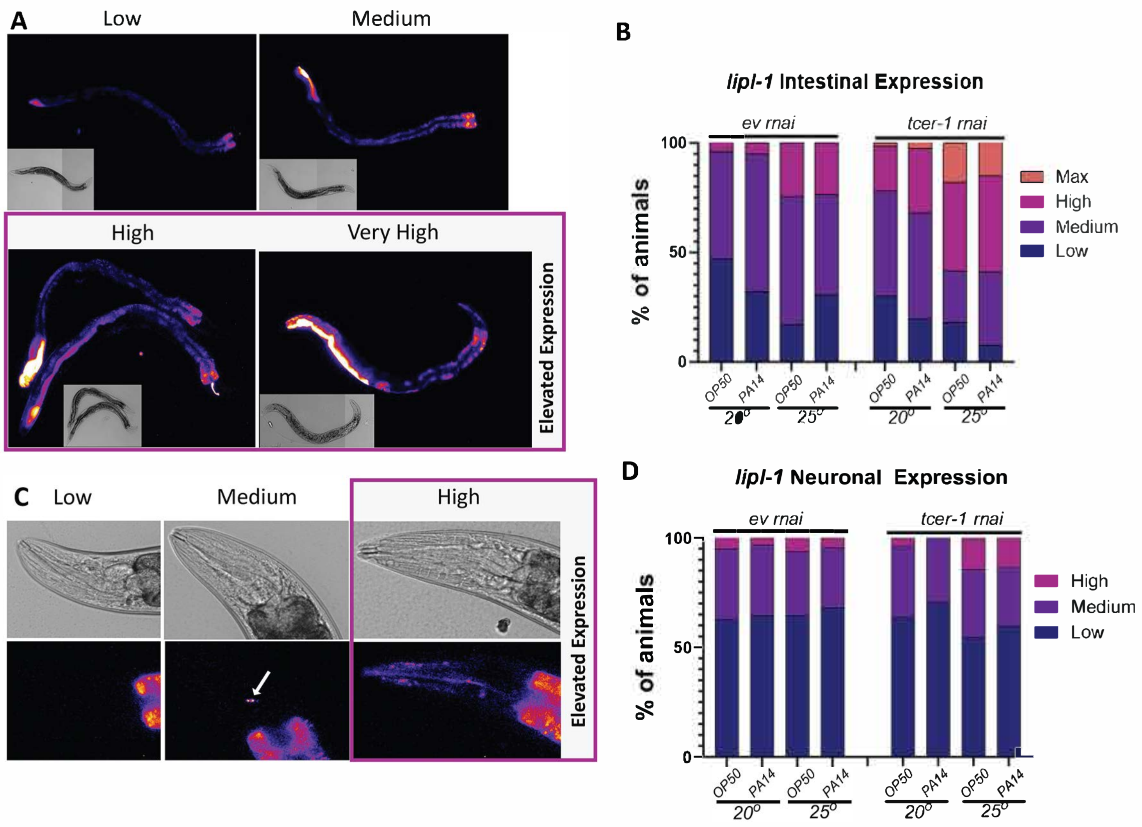

bioRxiv preprint doi: https://doi.org/10.1101/2025.07.14.664648; this version posted July 18, 2025. The copyright holder for this preprint (which was not certified by peer review) is the author/funder, who has granted bioRxiv a license to display the preprint in perpetuity. It is made available under aCC-BY 4.0 International license.

Supplementary Fig. S2: Categorical analysis of *Plipl-1::mCherry* expression. Flourescence levels quantified based on area and observable intensity variation (see Methods). **A, B: Intestinal Expression. A:** Representative images of each category pseudocolored with ImageJ LUT Fire. ***Low-*** dim fluorescence primarily visible in posterior and anterior intestine. No areas of high intensity, and posterior fluorescence limited to region near tail. ***Medium***- posterior fluorescence between vulva and tail. Some areas of intermediate intensity in anterior intensity. ***High***- posterior fluorescence uniformly extended up to the vulva or with multiple areas of bright intensity. ***Very High***– posterior intestine exhibited very bright fluorescence that extended into the anterior half beyond the vulva and intermediate fluorescence extended at least to the vulva. Purple boxes indicate categories quantified in Figure 2E, F. **B:** Quantification of percent of population in each category. Data from 3 pooled biological replicates. EV: Empty vector control. (ev RNAi till L4 then OP50, 20°C, n=81), (ev RNAi till L4 then PA14 20°C, n=62), (*tcer-1* RNAi till L4 then OP50, 20°C, n=83), *(tcer-1* RNAi till L4 then PA14 20°C, n=41), (ev RNAi till L4 then OP50, 25°C, n=82), (ev RNAi till L4 then PA14, 25°C, n=69), (*tcer-1* RNAi till L4 then OP50, 25°C, n=84), *(tcer-1* RNAi till L4 then PA14, 25°C, n=68) **C, D: Expression in Head Region. C:** Representative images of each category pseudocolored with ImageJ LUT Fire. ***Low-*** No flourescence visible. ***Medium-*** 1 or 2 puncta seen. ***High-*** More than 2 puncta, or diffuse, non-punctate expression. **D:** Quantification of data from 3 pooled biological replicates. (ev RNAi till L4 then OP50 20°C, n=81), (ev RNAi till L4 then PA14 20°C, n=62), (*tcer-1* RNAi till L4 then OP50 20°C, n=83), *(tcer-1* RNAi till L4 then PA14 20°C, n=41), (ev RNAi till L4 then OP50

bioRxiv preprint doi: https://doi.org/10.1101/2025.07.14.664648; this version posted July 18, 2025. The copyright holder for this preprint (which was not certified by peer review) is the author/funder, who has granted bioRxiv a license to display the preprint in perpetuity. It is made available under aCC-BY 4.0 International license.

25°C, n=82), (ev RNAi till L4 then PA14 25°C, n=69), (*tcer-1* RNAi OP50 25°C, n=84), (*tcer-1* RNAi PA14 25°C, n=67).

bioRxiv preprint doi: https://doi.org/10.1101/2025.07.14.664648; this version posted July 18, 2025. The copyright holder for this preprint (which was not certified by peer review) is the author/funder, who has granted bioRxiv a license to display the preprint in perpetuity. It is made available under aCC-BY 4.0 International license.

FIGURE S3

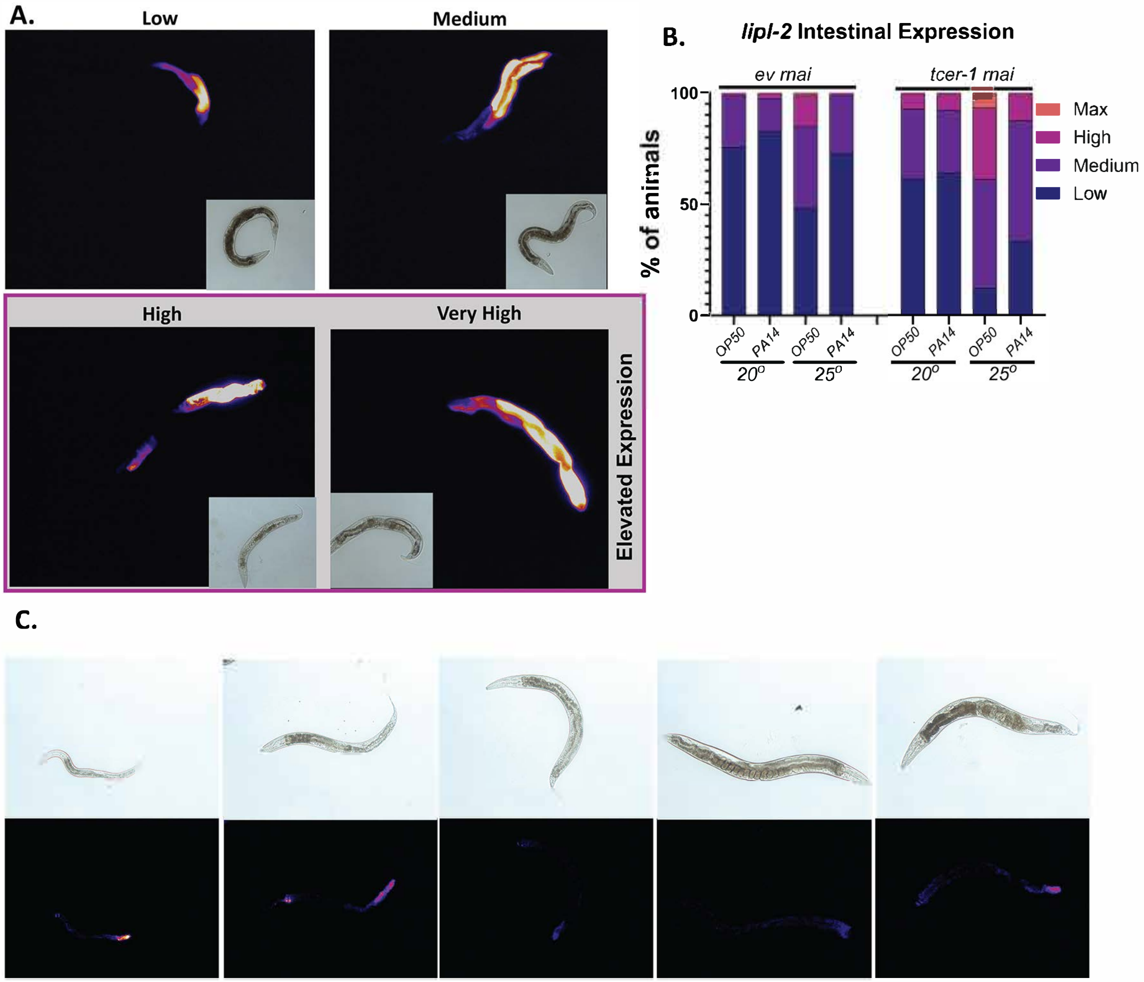

bioRxiv preprint doi: https://doi.org/10.1101/2025.07.14.664648; this version posted July 18, 2025. The copyright holder for this preprint (which was not certified by peer review) is the author/funder, who has granted bioRxiv a license to display the preprint in perpetuity. It is made available under aCC-BY 4.0 International license.

Supplementary Fig. S3: Categorical analysis of *Plipl-2::mCherry* expression. Expression levels in a population were variable and primarily observed in intestines. Flourescence quantified based on area and observable intensity variation (see Methods). **A, B: Intestinal Expression. A:** Representative images of each category pseudocolored with ImageJ LUT Fire. ***Low-*** dim fluorescence primarily visible in posterior intestine limited to region between vulva and tail. Brighter fluorescence, if present, was restricted to tail. ***Medium***- medium fluorescence in region between vulva and tail. Some area of increased intensity which extended past tail into anterior regions. ***High*** – broader posterior signal extending beyond vulva to anterior intestine with areas of bright intensity in posterior intestine. ***Very High***– Intense fluorescence extended from posterior intestine to anterior of vulva. Purple boxes indicate categories quantified in elevated expression analysis in Figure 2E, F. **B:** Quantification of percent of population in each category. Data from 3 pooled biological replicates. EV: Empty vectrol control. (ev RNAi till L4 then OP50 20°C, n=86), (ev RNAi till L4 then PA14 20°C, n=81), (*tcer-1* RNAi till L4 then OP50 20°C, n=83), *(tcer-1* RNAi PA14 till L4 then 25°C, n=78), (ev RNAi till L4 then OP50 25°C, n=87), (ev RNAi till L4 then PA14 25°C, n=81), (*tcer-1* RNAi till L4 then OP50 25°C, n=90), *(tcer-1* RNAi till L4 then PA14 25°C, n=72) **C: Expression dynamics across lifespan.** Representative images of *Plipl-2:*mCherry expression in (from left to right) young larvae, L4 larvae, young adult, Day 1 adult, and Day 5 adult worms. Pseudocolored in ImageJ with LUT Fire.

bioRxiv preprint doi: https://doi.org/10.1101/2025.07.14.664648; this version posted July 18, 2025. The copyright holder for this preprint (which was not certified by peer review) is the author/funder, who has granted bioRxiv a license to display the preprint in perpetuity. It is made available under aCC-BY 4.0 International license.

FIGURE S4

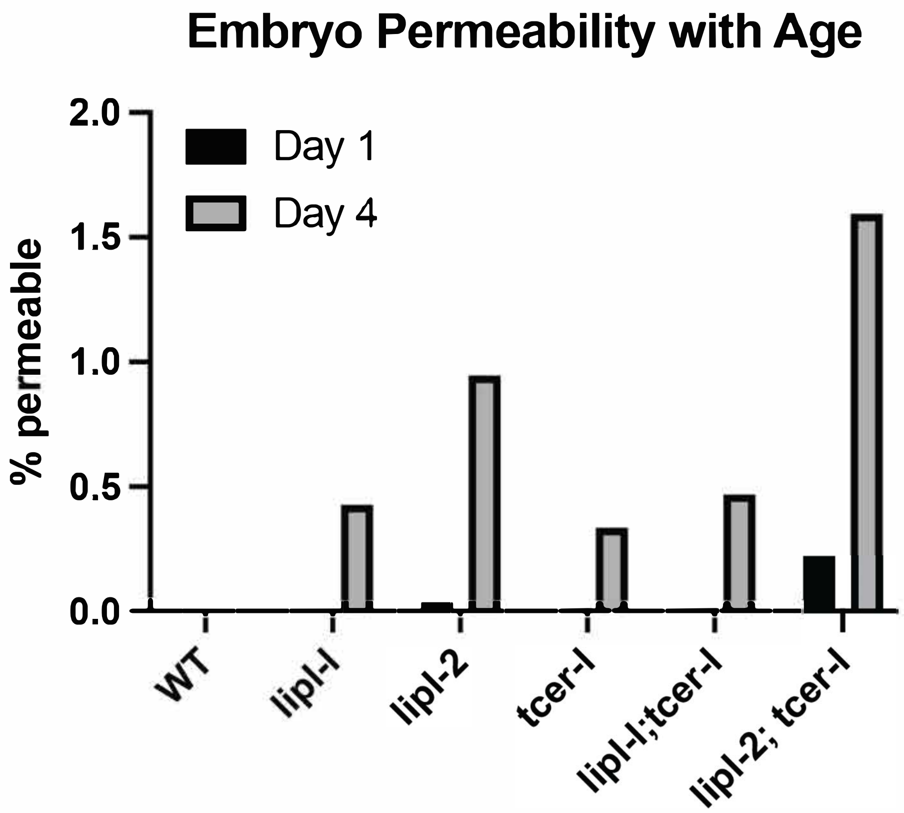

bioRxiv preprint doi: https://doi.org/10.1101/2025.07.14.664648; this version posted July 18, 2025. The copyright holder for this preprint (which was not certified by peer review) is the author/funder, who has granted bioRxiv a license to display the preprint in perpetuity. It is made available under aCC-BY 4.0 International license.

Supplementary Figure S4: Loss of *tcer-1*, *lipl-1* and *lipl-2* causes embryonic eggshell defects. Longitudinal analysis of BODIPY-permeable eggs laid by Day 1 (D1) and Day 4 (D4) mothers by different strains. WT (D1 0, n=712; D4 0, n=276), *lipl-l* (D1 0, n=700; D4 0.4267, n=703), *lipl-2* (D1 0.0356, n=2806; D4 0.9451, n=529), *tcer-l* (D1 0, n=896; D4 0.3361, n=596), *tcer-1;lipl-1* (D1 0, n=1456; D4 0.4687, n=640), *tcer-1;lipl-2* (D1 0.2212, n=1356; D4 1.594, n=439).

bioRxiv preprint doi: https://doi.org/10.1101/2025.07.14.664648; this version posted July 18, 2025. The copyright holder for this preprint (which was not certified by peer review) is the author/funder, who has granted bioRxiv a license to display the preprint in perpetuity. It is made available under aCC-BY 4.0 International license.

FIGURE S5

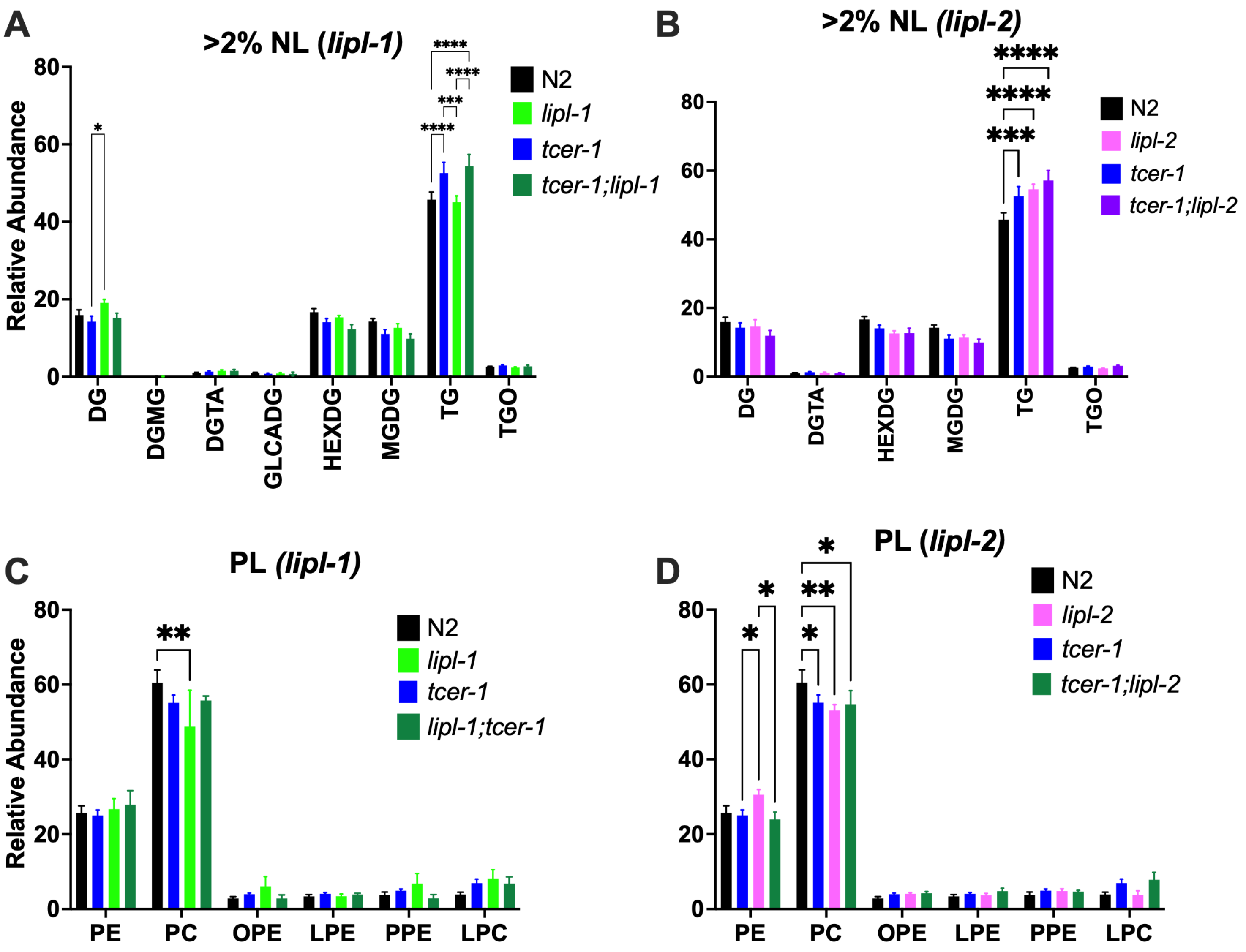

bioRxiv preprint doi: https://doi.org/10.1101/2025.07.14.664648; this version posted July 18, 2025. The copyright holder for this preprint (which was not certified by peer review) is the author/funder, who has granted bioRxiv a license to display the preprint in perpetuity. It is made available under aCC-BY 4.0 International license.

Supplementary Figure S5: Impact of *lipl-1* and *lipl-2* deletions on relative abundance of neutral lipid (NL) and phospholipid (PL) classes.

Lipids from gravid Day 1 adults were analyzed using HPLC-MS/MS. **A, B:** Impact of *lipl-1* (A) or *lipl-2* (B) inactivation on relative abundance of NLs with > 2% abundance in total NL population. **C, D:** Relative abundance of PL categories altered by *lipl-1* (C) or *lipl-2* (D) inactivation. Statistical significance was calculated using two-way ANOVA with Tukey's correction, p≤ 0.05(*), p < 0.01 (**), <0.001 (***), <0.0001 (****).

bioRxiv preprint doi: https://doi.org/10.1101/2025.07.14.664648; this version posted July 18, 2025. The copyright holder for this preprint (which was not certified by peer review) is the author/funder, who has granted bioRxiv a license to display the preprint in perpetuity. It is made available under aCC-BY 4.0 International license.

FIGURE S6

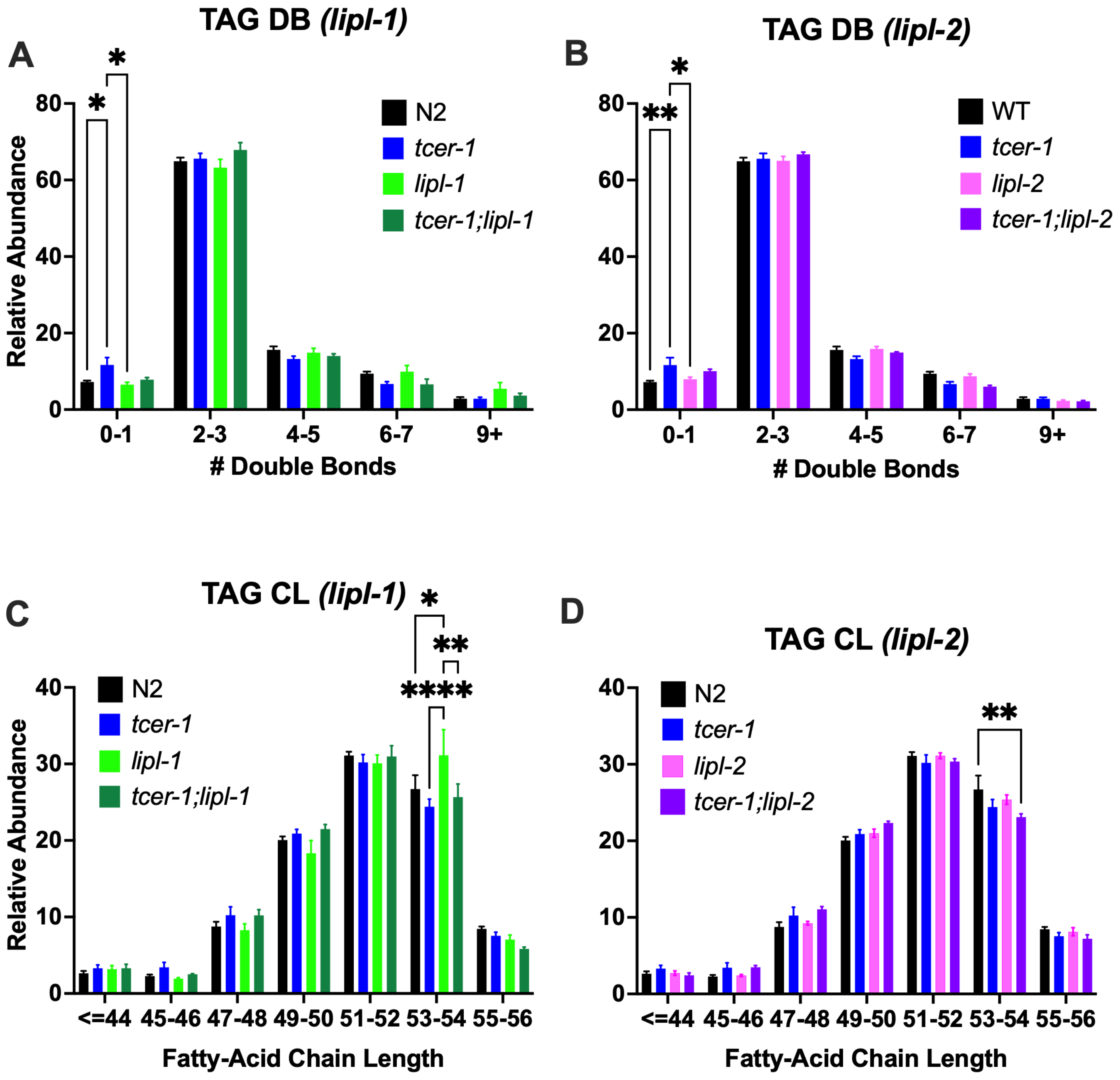

bioRxiv preprint doi: https://doi.org/10.1101/2025.07.14.664648; this version posted July 18, 2025. The copyright holder for this preprint (which was not certified by peer review) is the author/funder, who has granted bioRxiv a license to display the preprint in perpetuity. It is made available under aCC-BY 4.0 International license.

Supplementary Figure S6: Impact of *lipl-1* and *lipl-2* deletions on saturation and fatty-acid chain length.

A, B: Relative abundance of double bonds (DB) in triacylglycerides (TAG) altered by *lipl-1* (A) or *lipl-2* (B) inactivation. **C, D:** Relative abundance fatty-acid chain length (CL) of TAGs altered by *lipl-1* (C) or *lipl-2* (D) inactivation. Color key indicated on each panel. Statistical significance was calculated using two-way ANOVA with Tukey's correction, $p \leq 0.05$(*), $p < 0.01$ (**), <0.001 (***), <0.0001 (****).

bioRxiv preprint doi: https://doi.org/10.1101/2025.07.14.664648; this version posted July 18, 2025. The copyright holder for this preprint (which was not certified by peer review) is the author/funder, who has granted bioRxiv a license to display the preprint in perpetuity. It is made available under aCC-BY 4.0 International license.

FIGURE S7

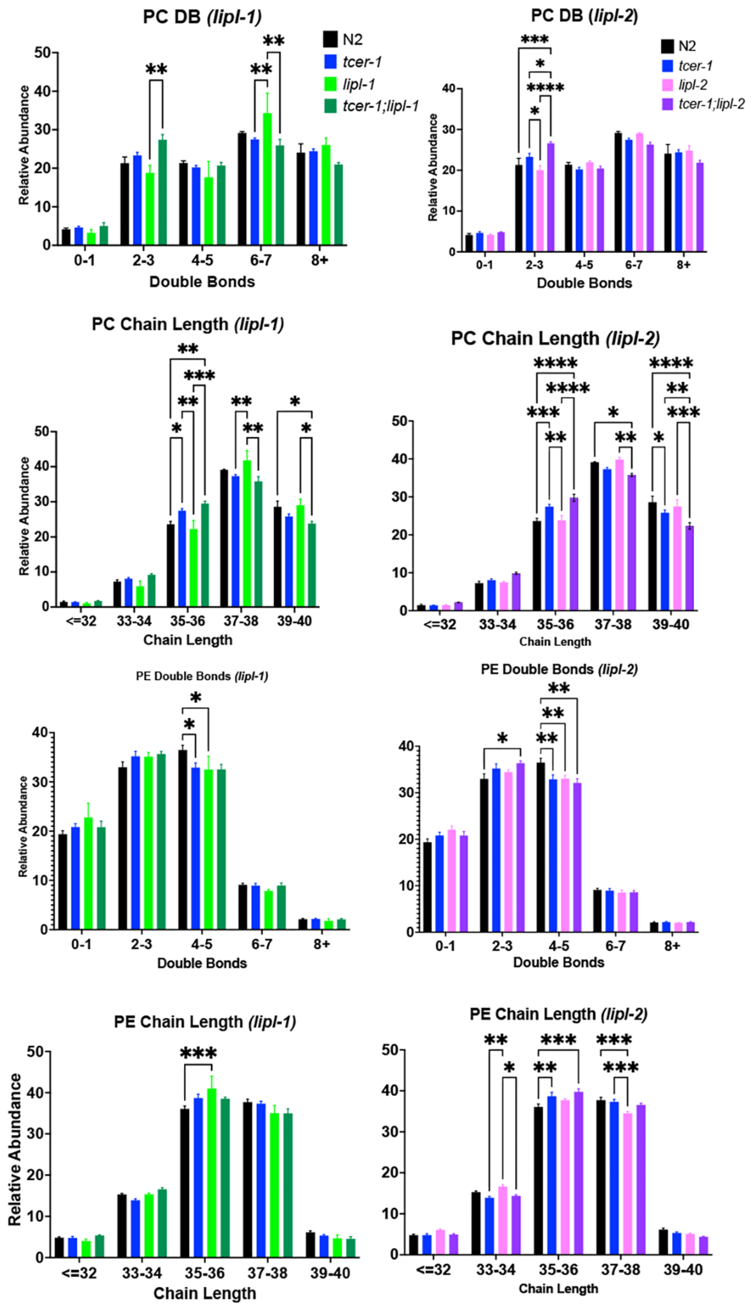

bioRxiv preprint doi: https://doi.org/10.1101/2025.07.14.664648; this version posted July 18, 2025. The copyright holder for this preprint (which was not certified by peer review) is the author/funder, who has granted bioRxiv a license to display the preprint in perpetuity. It is made available under aCC-BY 4.0 International license.

Supplementary Figure S7: Impact of *lipl-1* and *lipl-2* deletions on saturation and fatty-acid chain length of phosphatidylcholine (PC) and phosphatidyl-ethanolamine (PE).

A, B: Relative abundance of PC double bonds (~~DB~~) altered by *lipl-1* (A) or *lipl-2* (B) inactivation. **C, D:** Relative abundance of PC chain length (CL) altered by *lipl-1* (C) or *lipl-2* (D) inactivation. **E, F:** Relative abundance of PE double bonds altered by *lipl-1* (E) or *lipl-2* (F) inactivation. **G, H:** Relative abundance of PE chain length altered by *lipl-1* (G) or *lipl-2* (H) inactivation. Color key of different strains shown at top. Statistical significance was calculated using two-way ANOVA with Tukey's correction, $p \leq 0.05$(*), $p < 0.01$ (**), <0.001 (***), <0.0001 (****).

bioRxiv preprint doi: https://doi.org/10.1101/2025.07.14.664648; this version posted July 18, 2025. The copyright holder for this preprint (which was not certified by peer review) is the author/funder, who has granted bioRxiv a license to display the preprint in perpetuity. It is made available under aCC-BY 4.0 International license.

FIGURE S8

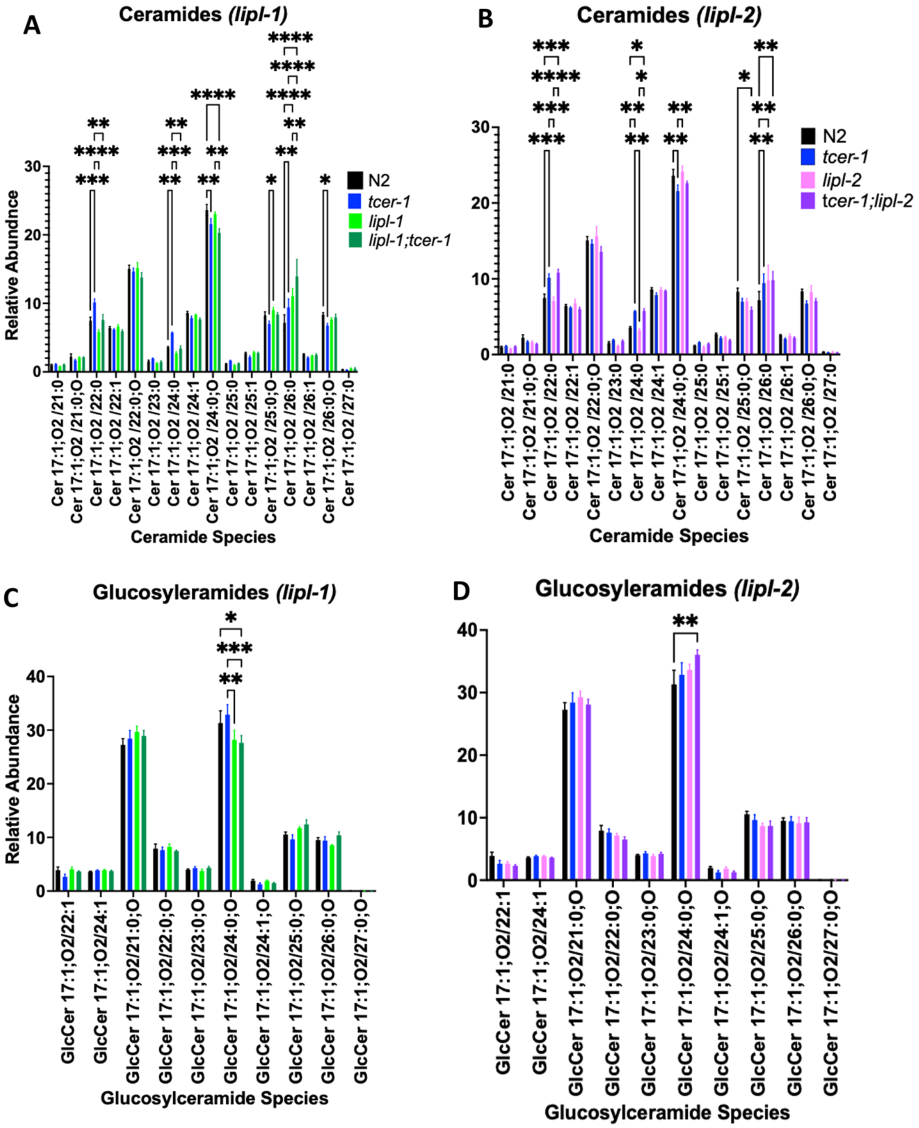

bioRxiv preprint doi: https://doi.org/10.1101/2025.07.14.664648; this version posted July 18, 2025. The copyright holder for this preprint (which was not certified by peer review) is the author/funder, who has granted bioRxiv a license to display the preprint in perpetuity. It is made available under aCC-BY 4.0 International license.

Supplementary Figure S8: Impact of *lipl-1* and *lipl-2* deletions on glucosyl-ceramides (GlcCer) and ceramides (Cer).

A, B: Relative abundance of GlcCers altered by *lipl-1* (A) or *lipl-2* (B) inactivation. **C, D:** Relative Abundance of Cers altered by *lipl-1* (C) or *lipl-2* (D) inactivation. Color key of different strains shown at top. Statistical significance was calculated using two-way ANOVA with Tukey's correction, p≤ 0.05 (*), p < 0.01 (**), <0.001 (***), <0.0001 (****).

bioRxiv preprint doi: https://doi.org/10.1101/2025.07.14.664648; this version posted July 18, 2025. The copyright holder for this preprint (which was not certified by peer review) is the author/funder, who has granted bioRxiv a license to display the preprint in perpetuity. It is made available under aCC-BY 4.0 International license.

List of Supplementary Tables

Table S1: Gene lists and GO-term analyses of differentially expressed genes identified by RNAseq in this study.

Table S2: Overlaps between PA14-induced genes identified in this study and previously reported PA14-driven genes.

Table S3: Survival of different strains upon PA14 infection.

Table S4: Impact of *lipl-1* and *lipl-2* null mutants on lifespan on *E. coli* OP50.

Table S5: Impact of *lipl-1* and *lipl-2* knockouts on survival upon *S. aureus* infection.

Table S6: Lipid species altered in *tcer-1* mutants and the impacts of *lipl-1* and *lipl-2* mutations on these alterations.

Table S7: Lipid species altered in *lipl-1* and *lipl-2* single mutants

Table S8: Survival of strains expressing human LAL (hLAL) in different genetic backgrounds.

Table S9: Strains used in this study.

Table S10: Primers used in this study.

bioRxiv preprint doi: https://doi.org/10.1101/2025.07.14.664648; this version posted July 18, 2025. The copyright holder for this preprint (which was not certified by peer review) is the author/funder, who has granted bioRxiv a license to display the preprint in perpetuity. It is made available under aCC-BY 4.0 International license.

Table S3: Impact of *lipl-1* and *lipl-2* null mutants on survival upon *P. aeruginosa* PA14 infection.

Strain	Background Genotype	n = obs/ total	Mean	SE ^	P (*vs* N2)	P (*vs tcer-1*)	P (*vs lipl-1*)
***lipl-1* Strains**							
		**Trial 1**			**Bonferroni P-value**		
N2	WT	75/98	49.72	0.86			
CF2166	*tcer-1*	91/147	64.03	1.57	**0.00000002**		
AGP347	*lipl-1*	59/119	52.07	1.4	**0.0184**		
AGP354	*tcer-1;lipl-1*	75/135	56.32	1.57	**0**	0.1229	
		**Trial 2**			**Bonferroni P-value**		
N2	WT	56/95	52.88	1.35			
CF2166	*tcer-1*	68/128	63.69	1.63	**0**		
AGP347	*lipl-1*	78/160	49.68	0.87	0.7954	**0.000000014**	
AGP354	*tcer-1;lipl-1*	101/126	48.17	0.8	1	**0**	
		**Trial 3**			**Bonferroni P-value**		
N2	WT	41/50	56.64	2.05			
CF2166	*tcer-1*	100/120	71.59	2.04	**0.0001**		
AGP347	*lipl-1*	104/110	54.06	1.14	0.152		
AGP354	*tcer-1;lipl-1*	51/64	58.17	1.9	1	**0.0002**	
		**Trial 4**			**Bonferroni P-value**		
N2	WT	76/93	52.58	1.41			
CF2166	*tcer-1*	68/100	66.56	1.55	**0.00000015**		
AGP347	*lipl-1*	77/94	56.29	1.19	1		
AGP354	*tcer-1;lipl-1*	73/95	59.76	1.34	0.1131	**0.0001**	
***lipl-2* Strains**							
**Strain**	**Background Genotype**	**Trial 5**			**Bonferroni P-value**		
		**n = obs/ total**	**Mean**	**SE ^**	**P (*vs* N2)**	**P (*vs tcer-1*)**	**P (*vs lipl-2*)**
N2	WT	102/119	59.27	1.21			
AGP364a	*lipl-2*	117/126	60.74	1.47	1		
AGP336a	*tcer-1*	98/123	67.44	1.51	**0**		
AGP358	*tcer-1;lipl-2*	84/127	75.27	1.85		**0.0045**	
AGP360a	*tcer-1; lipl-1 lipl-2*	124/138	55.56	1.34		**0**	**0**
		**Trial 6**			**Bonferroni P-value**		
N2	WT	113/148	63.79	1.25			
AGP364a	*lipl-2*	107/123	72.55	2.15	0.1846		
AGP336a	*tcer-1*	123/134	91.69	2.25	**0**		
AGP358	*tcer-1;lipl-2*	100/129	95.53	3.07		1	
AGP360a	*tcer-1; lipl-1 lipl-2*	107/140	67.58	1.71		**0**	**0**
		**Trial 7**			**Bonferroni P-value**		
	WT	86/113	51.13	1.32			vs. *lipl-1* /vs. *lipl-2*
AGP347	*lipl-1*	102/134	52.17	1.1	1		
AGP364a	*lipl-2*	61/123	70.64	1.5	**0**		
AGP357a	*lipl-1 lipl-2*	83/117	51.85	1.03	1		0.7811/**0**
AGP336a	*tcer-1*	87/112	70.61	2.09	**0**		
AGP354	*tcer-1;lipl-1*	85/120	56.04	1.23	**0.0004**	**0.0009**	
AGP358	*tcer-1;lipl-2*	66/124	78.23	2.66		1	
AGP360a	*tcer-1; lipl-1 lipl-2*	78/128	53.99	1.3		**0.000029**	**0.000024/0**

bioRxiv preprint doi: https://doi.org/10.1101/2025.07.14.664648; this version posted July 18, 2025. The copyright holder for this preprint (which was not certified by peer review) is the author/funder, who has granted bioRxiv a license to display the preprint in perpetuity. It is made available under aCC-BY 4.0 International license.

Table S4: Impact of *lipl-1* and *lipl-2* null mutants on lifespan on *E. coli* OP50.

Strain	Background Genotype	Trial 1			Bonferroni P-value	
		n = obs/ total	Mean	SE ^	P (*vs* N2)	P (*vs tcer-1*)
N2	WT	77/121	19.03	0.77		
AGP347	*lipl-1*	79/116	17.4	0.65	0.537	
AGP364a	*lipl-2*	71/120	20.6	0.56	1	
AGP357	*lipl-1 lipl-2*	53/124	18.1	0.74	1	
AGP336a	*tcer-1*	65/118	15.87	0.74	0.0665	
AGP354	*tcer-1;lipl-1*	72/115	19.36	0.79		**0.0113**
AGP358	*tcer-1;lipl-2*	87/117	19.19	0.78		**0.0297**
AGP360a	*tcer-1;lipl-1 lipl-2*	82/117	15.75	0.5		1
**Trial 2**						
N2	WT	55/103	15.75	0.68		
AGP347	*lipl-1*	71/110	13.81	0.67	1	
AGP364a	*lipl-2*	52/103	15.8	0.96	1	
AGP357	*lipl-1 lipl-2*	64/100	11.95	0.66	**0.0004**	
AGP336a	*tcer-1*	39/103	12.08	0.76	**0.0031**	
AGP354	*tcer-1;lipl-1*	83/103	12.9	0.54		1
AGP358	*tcer-1;lipl-2*	79/97	15.31	0.69		**0.0369**
AGP360a	*tcer-1;lipl-1 lipl-2*	80/104	13.78	0.72		0.5519
**Trial 3**						
N2	WT	94/121	14.96	0.34		
AGP347	*lipl-1*	80/106	15.79	0.57	0.2334	
AGP364a	*lipl-2*	89/113	15.93	0.56	0.1141	
AGP357	*lipl-1lipl-2*	101/114	13.84	0.48	1	
AGP336a	*tcer-1*	92/120	14.23	0.48	1	
AGP354	*tcer-1;lipl-1*	77/120	15.36	0.51		0.9902
AGP358	*tcer-1;lipl-2*	81/103	16.72	0.58		**0.0133**
AGP360a	*tcer-1;lipl-1 lipl-2*	86/114	13.84	0.35		1

bioRxiv preprint doi: https://doi.org/10.1101/2025.07.14.664648; this version posted July 18, 2025. The copyright holder for this preprint (which was not certified by peer review) is the author/funder, who has granted bioRxiv a license to display the preprint in perpetuity. It is made available under aCC-BY 4.0 International license.

Table S5: Impact of *lipl-1* and *lipl-2* knockouts on survival upon *S. aureus* infection.

Genotype	# Animals	Mean (Hours)	S.E.M	P-value		Bonferroni P-value	
				vs. WT	*vs. tcer-1*	vs. WT	*vs. tcer-1*
**Trial 1**							
**WT**	65	40.26	1.47				
tcer-1	60	53.45	1.75	<0.0001		<0.0001	
tcer-1; lipl-1	71	49.19	1.54	<0.0001	0.95	<0.0001	1
tcer-1;lipl-2	43	57.42	2.2	<0.0001	0.03	<0.0001	0.08
**Trial 2**							
WT	81	34.84	1.25				
tcer-1	76	59.21	2.33	<0.0001		<0.0001	
tcer-1; lipl-1	65	52.72	2.08	<0.0001	0.16	<0.0001	0.83
tcer-1;lipl-2	63	64.95	2.43	<0.0001	0.28	<0.0001	0.49
**Trial 3**							
**WT**	73	41.07	1.90				
lipl-1	73	42.29	1.82	0.01	<0.0001	0.02	<0.0001
tcer-1	60	50.50	2.29	<0.0001		<0.0001	
tcer-1; lipl-1 lipl-2	80	40.14	1.82	0.51	<0.0001	1.00	<0.0001
**Trial 4**							
**WT**	59	**33.62**	1.71				
lipl-1	41	**41.32**	2.49	0.81	0.18	1	0.53
tcer-1	43	**50.56**	3.08	0.02		0.05	
tcer-1; lipl-1 lipl-2	50	**45.61**	2.29	0.08	<0.0001	0.24	<0.0001
**Trial 5**							
**WT**	72	43.16	1.82				
lipl-1	65	40.99	2.12	0.15	0.32	0.44	0.75
tcer-1	52	46.10	2.50	0.64		1.00	
tcer-1; lipl1 lipl2	72	40.45	1.38	0.89	0.02	1.00	0.06
**Trial 6**							
**WT**	73	39.05	2.47				
lipl-2	73	54.35	2.46	<0.0001		<0.0001	
lipl-1 lipl-2	77	56.32	1.74	<0.0001		<0.0001	
**Trial 7**							
**WT**	79	37.01	1.20				
lipl-2	74	54.25	2.00	<0.0001			
**Trial 8**							
**WT**	81	58.45	1.38				
lipl-2	58	64.06	1.11	<0.0001		<0.0001	
lipl-1 lipl2	76	61.58	1.41	0.01		0.02	
**Trial 9**							
**WT**	78	50.53	1.57				
lipl-1 lipl2	75	59.75	1.48	<0.0001		<0.0001	

bioRxiv preprint doi: https://doi.org/10.1101/2025.07.14.664648; this version posted July 18, 2025. The copyright holder for this preprint (which was not certified by peer review) is the author/funder, who has granted bioRxiv a license to display the preprint in perpetuity. It is made available under aCC-BY 4.0 International license.

Table S6: Lipid species altered in *tcer-1* mutants and impacts of *lipl-1* and *lipl-2* mutations on them.

Lipid Categories			WT vs *tcer-1*		*tcer-1;lipl-1* vs *tcer-1*		*tcer-1;lipl-2* vs *tcer-1*	
	**Major Class**	**Species**	***tcer-1* /WT Ratio**	**Statistical significance (q value)**	***tcer-1;lipl-1* /*tcer-1* Ratio**	**Statistical significance (q value)**	***tcer-1;lipl-2* /*tcer-1* Ratio**	**Statistical significance (q value)**
**Neutral Lipids**	**TG**		1.15	****	no significant changes			
	**MGDG**	MGDG 32:2	1.565737052	0.007177	no significant changes		no significant changes	
		MGDG 38:5	0.616957606	0.007177				
		MGDG 38:4	0.483273056	0.026845				
		MGDG 36:11	0.910572503	0.027074				
	**HEXDG**	HEXDG 37:2	0.464132231	0.003409	no significant changes		no significant changes	
		HEXDG 37:7	1.417880795	0.003409				
		HEXDG 37:9	0.750509857	0.005333				
		HEXDG 37:10	0.451458753	0.030091				
		HEXDG 37:4	0.714666021	0.034491				
**Phospho-lipids**	**PC**	PC 37:6	0.711318272	0.000803	no significant changes		no significant changes	
		PC 39:4	0.473088837	0.001716				
		PC 38:7	1.42758289	0.001716				
		PC 39:6	0.620129221	0.00195				
		PC 38:2	0.487076948	0.003067				
		PC 33:2	1.582312075	0.005082				
		PC 36:4	1.553013469	0.006053				
		PC 36:3	1.771081002	0.006053				
		PC 37:4	0.649282564	0.006981				
		PC 39:5	0.631199353	0.006981				
		PC 32:2	1.43658948	0.008009			1.782691575	0.00098
		PC 38:4	0.832043425	0.02792			no significant changes	
	**LPC**	LPC 18:2	1.653066306	0.000388	no significant changes		1.292298424	0.018886
		LPC 17:1	0.678693403	0.001427			no significant changes	
		LPC 19:1	0.538062535	0.001427				
	**PE**	PE 39:6	0.533642249	0.001592	no significant changes		no significant changes	
		PE 40:4	0.546087105	0.001592				
		PE 36:2	1.309779671	0.015054				
		PE 37:6	0.734816912	0.021557				
		PE 36:1	1.355844676	0.02955				
		PE 36:4	0.838629509	0.034792				
		PE 35:4	0.785869456	0.038881				
		PE 40:2	1.911385285	0.038881				
		PE 34:3	0.75335599	0.038881				
		PE 37:4	0.814092018	0.043916				
**Sphingo-lipids**	**Cer**	**Cer 17:1;O2 /22:0**	**1.355226491**	**0.0002**	**0.743967625**	**0.0032**	no significant changes	
		**Cer 17:1;O2 /24:0**	**1.613143178**	**0.0036**	**0.589059338**	**0.0097**		
		Cer 17:1;O2 /24:0; O	0.912544262	0.0065	no significant change			
		**Cer 17:1;O2 /26:0**	**1.310880231**	**0.0026**	**1.478907888**	**<0.0001**		
		Cer 17:1;O2 /26:0;O	0.804705029	0.0496	no significant change			
	**GlcCer**	no significant species changes						
		* p-value						

bioRxiv preprint doi: https://doi.org/10.1101/2025.07.14.664648; this version posted July 18, 2025. The copyright holder for this preprint (which was not certified by peer review) is the author/funder, who has granted bioRxiv a license to display the preprint in perpetuity. It is made available under aCC-BY 4.0 International license.

Table S7: Lipid species altered in *lipl-1* and *lipl-2* mutants

Lipid Categories			WT vs *lipl-1*		WT vs *lipl-2*	
	**Major Class**	**Species**	***lipl-1* /WT Ratio**	**Statistical significance (q value)**	***lipl-2* /WT Ratio**	**Statistical significance (q value)**
**Neutral Lipids**	**TG**	no significant changes				
	**MGDG**	no significant changes				
	**HEXDG**	no significant changes				
**Phospho-Lipids**	**PC**	no significant changes				
	**LPC**	no significant changes				
	**PE**	no significant changes				
**Sphingo- Lipids**	**Cer**	Cer 17:1;O2 /26:0	1.534223979	<0.0001	**1.360074367**	**0.0032**
		Cer 17:1;O2 /22:0	no significant changes		no significant changes	
	**GlcCer**	no significant changes				

bioRxiv preprint doi: https://doi.org/10.1101/2025.07.14.664648; this version posted July 18, 2025. The copyright holder for this preprint (which was not certified by peer review) is the author/funder, who has granted bioRxiv a license to display the preprint in perpetuity. It is made available under aCC-BY 4.0 International license.

Table S8: Survival of strains expressing human LAL (hLAL) in different genetic backgrounds.

Strain	Genotype	Trial 1			Bonferroni P-value		
		n = obs/ total	Mean	SE ^	P (vs WT)	*P (vs tcer-1)*	*P (vs. tcer-1;lipl-1)*
WT*	WT	54/90	35.06	1.91		**0.0000034**	1
CF2166*	*tcer-1*	63/90	54.8	3.25	**0.0000034**		**0.000021**
AGP354*	*tcer-1;lipl-1*	52/90	36.65	1.79	1	**0.000021**	
*	*tcer-1;lipl-1;hLAL (A)*	73/90	55.77	2.53	**0**	1	**0**
	tcer-1;lipl-1;hLAL (B)	70/90	40.14	1.68	0.0738	**0.0002**	0.5904
**Trial 2**							
N2	WT	51/90	40.29	2.7		**0**	**0.0000027**
CF2166	*tcer-1*	54/90	68.56	2.99	**0**		**0.0126**
AGP354	*tcer-1;lipl-1*	42/90	55.31	4.57	**0.0000027**	**0.0126**	
	tcer-1;lipl-1;hLAL (A)	43/90	63.53	2.59	**0**	0.4523	0.0866
	tcer-1;lipl-1;hLAL (B)	39/90	63.08	3.66	**0**	0.6723	0.1765

** Shown in Figure 7A*

bioRxiv preprint doi: https://doi.org/10.1101/2025.07.14.664648; this version posted July 18, 2025. The copyright holder for this preprint (which was not certified by peer review) is the author/funder, who has granted bioRxiv a license to display the preprint in perpetuity. It is made available under aCC-BY 4.0 International license.

Table S9: Strains used in this study.

Strain name	Genotype	Transgene/Description	Description/Comment	Source
N2	WT			
AGP334	N2	*[Plipl-1(441bp)::mCherry + Pmyo-3::GFP] 50ng/ul, 15ng/ul*	mCherry driven under 441bp *lipl-1* promoter	This study
AGP340	N2	*[Plipl-1(1kb)::mCherry + Pmyo-3::GFP] 50ng/ul, 15ng/ul*	mCherry driven under 1016bp *lipl-1* promoter	This study
AGP335	N2	*[Plipl-2(1.5kb)::mCherry + Pofm-1::GFP] 50ng/ul, 15ng/ul*	mCherry driven under 1.5kb *lipl-2* promoter	This study
AGP342	N2	*[Plipl-2(1kb)::mCherry + Pmyo-3::GFP] 50ng/ul, 50ng/ul*	mCherry driven under 1kb *lipl-2* promoter	This study
AGP341	N2	*[Plipl-2::LIPL-2::mRFP + Pmyo-3::GFP] 25ng/ul, 15ng/ul*	LIPL-2::RFP overexpression	This study
AGP339	N2	*[Plipl-1(1kb)::LIPL-1::mRFP + Pmyo-3::GFP] 25ng/ul, 15ng/ul*	LIPL-1::RFP overexpression	This study
COP2589	EG6699/COP93	*knuSi924 [pNU3447 (eft-3p::hLIPA::linker::wrmScarlet::3xFLAG::tbb-2u in cxTi10882, unc-119(+))] IV ; unc-119(ed3) III*	Human LAL expressed broadly in soma	This study
COP2593	EG6699/COP93	*knuSi927 [pNU3447 (eft-3p::hLIPA::linker::wrmScarlet::3xFLAG::tbb-2u in cxTi10882, unc-119(+))] IV ; unc-119(ed3) III*	Human LAL expressed broadly in soma	This study
VS56	N2	*hj340 [cpg-2 signal peptide::mCherry_TEV_3xFLAG::cpg-2] ltIs38 [pie-1p::GFP::PH(PLC1delta1) + unc-119(+)]*	mCherry::CPG-2 and GPF::PH(PLC1delta1) ex	gift from Dr. Ho Yi Mak
CF2166	*tcer-1*	*tcer-1(tm1452) II*		
AGP336a	*tcer-1*	*tcer-1(tm1452)/mnC1 [dpy-10(e128) unc-52(e444)] II.*		This study
AGP347	*lipl-1*	*full crispr deletion*		This study
AGP364a	*lipl-2*	*full crispr deletion*		This study
AGP357a	*lipl-1;lipl-2*	*full crispr deletion both lipases*		This study
AGP354	*tcer-1;lipl-1*	*tcer-1(tm1452) crossed to lipl-1 Crisper null*		This study
AGP358	*cer-1;lipl-2*	*tcer-1(tm1452) crossed to lipl-2Crisper null*		This study
AGP360a	*tcer-1; lipl-1 lipl-2*	*tcer-1(tm1452) crossed to lipl-1 lipl-2 Crisper double null*		This study
AGP366	*tcer-1; hLAL*	*COP293 crossed into tcer-1(tm1452) mutant*		This study
AGP367a	*lipl-1; hLAL*	*COP293 crossed into lipl-1 Crisper null mutant*		This study
AGP368b	*tcer1;lipl-1;hLAL*	*COP293 crossed into tcer-1;lipl-1 mutant strain*		This study

bioRxiv preprint doi: https://doi.org/10.1101/2025.07.14.664648; this version posted July 18, 2025. The copyright holder for this preprint (which was not certified by peer review) is the author/funder, who has granted bioRxiv a license to display the preprint in perpetuity. It is made available under aCC-BY 4.0 International license.

Table S10: Primers used in this study.

Gene	direction	Sequence
**Diagnostic Genotyping**		
lipl-2	F	TGCACAAAGAGTTTGGCGTT
	R1	TCCCCATCGTTCAATGATTTGACT
	R2	AGTCCGGTTGATGCGACAAA
lipl-1	F	CCGTGACTGCGTTTTTCGTT
	R1	GACCTGGGGCACTCACTTTT
	R2	TGTGTTAACCGTGTGACTCCT
tcer-1	F	gccaattctggttgagtgac
	R	TCGTCTTGACTGATGGACAC
**QPCR**		
lipl-1	F	GGACTTAAAGTTGAAGCTGGAG
	R	AACACGAGTTGCGTTAAGC
lipl-2	F	GTTACATGGCCAAATGGGA
	R	AAACGAAAGCTGCACTCTG
rpl-32	F	GGATTTGGACATGCTCCTC
	R	GATTCCCTTGCGGCTCTT
Y45F10D.4	F	TTCACTGTTCAATGCTCGC
	R	CTTAGGCCTTCTTAGTCTGCT
**Cloning**		
lipl-2 1.5kb	F	gattacgccaagcttgcatgactatgagggccgtttcaattc
	R	cctcgcccttgctcaccatgtttgttctagtacgatcagttatcattac
lipl-1 441 kb	F	gattacgccaagcttgcatggtggaaatatttcagaaactttc
	R	cctcgcccttgctcaccatgttcaaccagagtctacctg
lipl-1 1kb	F	gattacgccaagcttgcatgaaaattgaaactttcgccac
	R	cctcgcccttgctcaccatgttcaaccagagtctacctg
lipl-2 1kb	F	gattacgccaagcttgcatgAATCATGAAAGTATAATAATTGAGG
	R	cctcgcccttgctcaccatgTTTGTTCTAGTACGATCAG
lipl-1::RFP	F	actatagggcgaattggggtaccagcttcaataaaaattgaaactttc
	R	tcggaggaggccatcccgggggaaccgttcaaaac
**CRISPR-Cas9 Oligos**		
dpy-10 co-CRISPR	repair template 1	CACTTGAACTTCAATACGGCAAGATGAGAATGACTGGAAACCGTACCGCAT
	repair template 2	GCGGTGCCTATGGTAGCGGAGCTTCACATGGCTTCAGACCAACAGCCTAT
	gRNA	GCUACCAUAGGCACCACGAGGUUUUAGAGCUAUGCU
lipl-1 target gRNA	F	AUUGUGCACAAAUUAAGGACGUUUUAGAGCUAUGCU
	R	CAUUAUUAUGUUCUUUUAACGUUUUAGAGCUAUGCU
	repair template	ATGGGCGGGCACAGTAATAATTGTGCACAAATTAAAAAGAACATAATAATGTTCTCAAAAT GTTATTTTA
lipl-2 target gRNA	F	GUUUAAGACUACAAUGUACUGUUUUAGAGCUAUGCU
	R	ACAGCUAAGGAUUAGAUCAUGUUUUAGAGCUAUGCU
	repair template	CTTCTAAATTCAGAGAAAATCTAAATTCTCCCCAAGTCATTGGAAAAGGAAGCAATGACAA AAATAGATCAC
